# Sphingosine-1-phosphate receptor 2 inhibition ameliorates familial exudative vitreoretinopathy models

**DOI:** 10.1016/j.jbc.2025.111107

**Published:** 2025-12-23

**Authors:** Hirad A. Feridooni, Rachel Fody, Mahtab Tavasoli, Mariam Alkandari, Sarah van der Ende, Michel Nader, Johane M. Robitaille, Christopher R. McMaster

**Affiliations:** 1 Department of Pharmacology, Dalhousie University, Halifax, Nova Scotia, Canada; 2 Faculty of Medicine, Dalhousie University, Halifax, Nova Scotia, Canada; 3 Departments of Ophthalmology and Visual Sciences, Pathology, Pediatrics, and Surgery Dalhousie University/IWK Health Centre, Halifax, Nova Scotia, Canada

**Keywords:** lipid, sphingolipid, signaling, endothelial cells, vascularization, inherited disease, ocular

## Abstract

Familial exudative vitreoretinopathy (FEVR) is an inherited childhood blinding disorder with close to 85% of molecularly determined cases due to rare variants in the genes encoding members of the frizzled 4 (FZD4) receptor complex. FEVR causes blindness due to complications arising from developmental peripheral non-perfusion of the retina. We sought to find a small molecule that could ameliorate FEVR models. In this study, we determine that the sphingosine1-phosphate receptor 2 (S1PR2) antagonist JTE-013 can ameliorate cellular and mouse models of FEVR. Using human primary retinal microvascular endothelial cells (hRMECs), we show that either knockdown of FZD4 expression using shRNA or expression of a known FEVR-causing dominant negative allele of FZD4 decreases the ability of hRMECs to tubularize. The addition of JTE-013 to both hRMEC models of FEVR resulted in the restoration of tubularization. In the well-established *Fzd4*^*−/−*^ mouse model of FEVR, dosing animals with JTE-013 ameliorated the retinal vascularization defects in these mice. The implications of these findings are (i) a major contributor to abnormal retinal angiogenesis in FEVR is likely through a decrease in vascular formation/integrity, and (ii) treatment with a well-characterized S1PR2 inhibitor restores normal vascularization in cell and mouse models of FEVR. To our knowledge, this is the first study to implicate S1PR signaling in FEVR and to show that a small drug-like molecule can restore normal vascularization and prevent blinding complications.

Familial exudative vitreoretinopathy (FEVR) is an inherited childhood blinding disorder characterized by an inability to vascularize the periphery of the retina resulting in visual impairment ranging from near-normal vision to complete blindness (1, 2). Within the first decade, FEVR can progress with development of aberrant neovascularization of the retina that causes retina tearing or detachment and complete blindness (Fig. 1) (3, 4, 5). Current FEVR treatment is laser surgery to ablate the non-vascularized area to prevent aberrant neovascularization; however, this does not improve peripheral vision and leaves patients with their impaired level of vision at birth and lifelong risks of retinal detachment (3).

**Figure 1:**
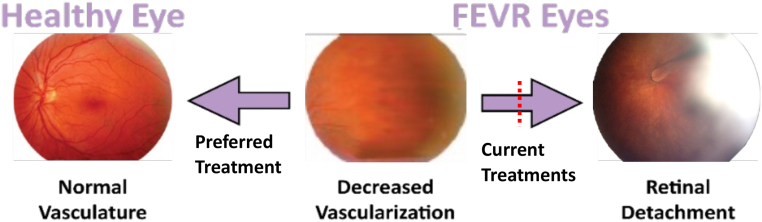
**Familial exudative vitreoretinopathy.** Decreased retinal vascularization is present in patients with familial exudative vitreoretinopathy (FEVR) at birth and can progress to aberrant neovascularization and retinal tearing and detachment, causing complete blindness. The image on the right depicts a retinal detachment in the form of a fold extending from the optic nerve to the lens of the eye, as well as severe generalized absence of normal retinal blood vessels. Current FEVR treatment is to ablate the non-vascularized area of the retina to prevent triggering of neovascularization and subsequent blinding complications. There is currently no treatment for FEVR that restores normal vasculature to the retina.

Approximately 85% of patients with molecularly defined FEVR (OMIM #133780) are due to a defect in the four genes that comprise the frizzled 4 (FZD4) receptor complex (2, 6, 7, 8, 9, 10, 11, 12). FZD4 (frizzled 4 receptor) is bound by its circulating ligand Norrin (*NDP*, the ligand for FZD4) and recruitment of the integral membrane proteins TSPAN12 and LRP5 to form the FZD4 receptor complex (2, 4, 13, 14, 15, 16, 17, 18, 19, 20, 21). It is thought that activation of the FZD4 receptor complex by Norrin results in β-catenin translocation to the nucleus to activate genes that increase retinal angiogenesis and vascular integrity, although this process is poorly understood (7, 13, 15, 16, 17, 18, 19, 20, 21, 22, 23, 24). FEVR can be inherited in an autosomal dominant or recessive fashion for *FZD4*, *TSPAN12*, and *LRP5*, while *NDP* is X-linked recessive. Estimations for FEVR prevalence range from 1:10,000 in early gene discovery papers to as high as 1:225 in a multi-center review of 62,799 newborns who had eye examinations using wide-field digital imaging within 28 days of birth (2, 25).

Vascular endothelial cells drive angiogenesis and subsequently form the inner layer of blood vessels. In mice, as in humans, retinal vasculature is initiated by the formation of a primary retinal vascular endothelial cell bed that extends from the optic nerve to the peripheral retina. Humans normally exhibit fully formed retinal vessels by term birth (3, 5). In mice, the primary vascular plexus develops in the first postnatal week, with the formation of the deeper layers completed by postnatal day 14 (P14), offering an opportunity to study the development of the retina and retinal vascular disorders (13, 15, 17, 18, 20).

An important signaling pathway that is implicated in vascular development and integrity is the sphingosine-1-phosphate (S1P) signaling pathway. The circulating lipid S1P regulates angiogenesis and vascular integrity by binding to S1P receptors (S1PR) (26, 27, 28, 29, 30, 31, 32, 33, 34, 35, 36, 37). The binding of S1P to S1PR1 or S1PR3 promotes angiogenesis and vascular integrity while S1P binding to S1PR2 inhibits these processes. Specifically, S1PR1 primarily couples to the G_i_ family of G proteins with subsequent downstream activation of the PI3K/Akt/Rac pathway, promoting the assembly and stabilization of adherens and tight junctions, preventing gaps between adjacent endothelial cells and stabilizing the developing vasculature (26, 27, 28, 35, 36, 38). The S1PR2 is primarily coupled to G_12/13_ to activate the Rho-Rac kinase pathway, which inhibits endothelial cell migration, tubule formation, and angiogenesis (27, 34, 36, 37). S1PR3 is primarily coupled to G_q_ and activates phospholipase C which is believed to promote endothelial cell migration and proliferation (26, 27, 28, 29, 30, 35, 36, 37, 38).

There is currently no treatment, or indeed an alternate pathway whose activity can be modified, that can promote revascularization of the retina in patients with FEVR to maintain peripheral vision and eliminate secondary blinding complications. In this study, we sought to test whether inhibition of S1PR2 signaling could restore retinal vasculature in FEVR models and determine the biological process(es) required.

## Results

### FZD4 inhibition disrupts retinal endothelial tubulogenesis

**Figure 2:**
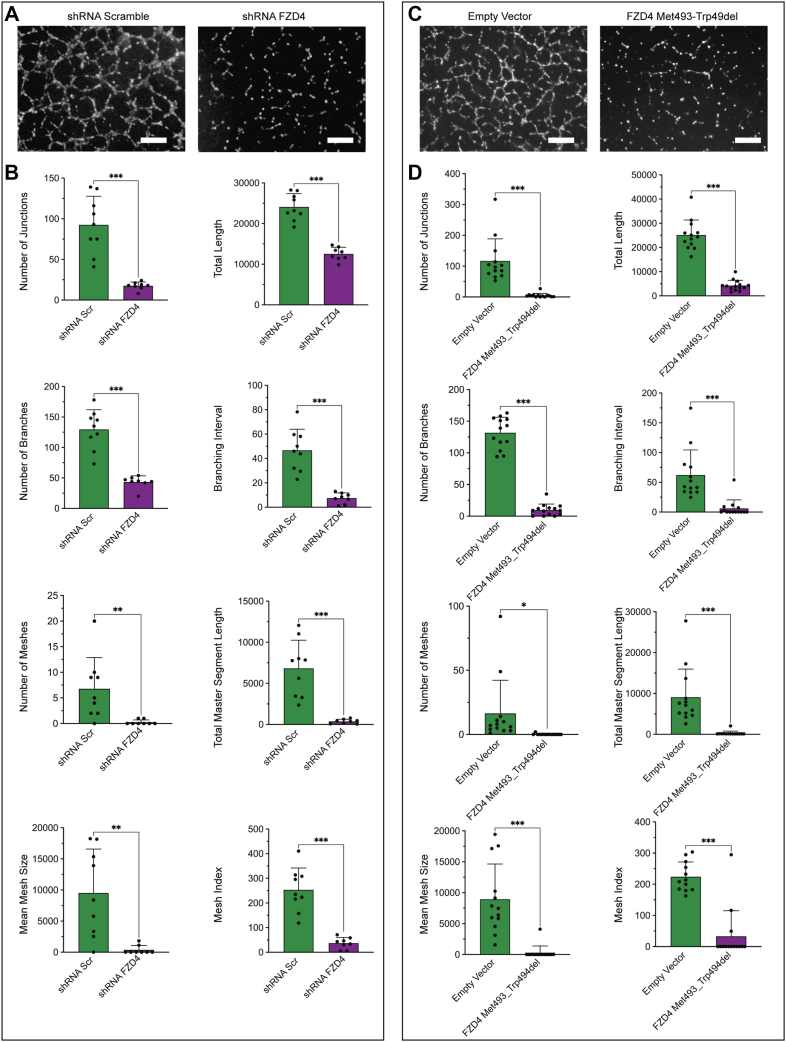
***FZD4* inhibition prevents primary human retinal microvascular endothelial cell (hRMEC) tubularization.***A*, Microscopy of tubularization in primary hRMECs treated with *FZD4* shRNA. *B*, computational analysis of vascularization in both *FZD4* shRNA treated and control. Eight different parameters were examined and measured using FIJI (Table S1, Fig. S2). *C*, microscopy of tubularization in hRMECs due to expression of the patient derived dominant-negative FZD4 Met493-Trp49del allele. *D*, computational analysis of vascularization in both overexpression of the *FZD4* variant and the control. There is significant reduction in all eight examined parameters when the *FZD4* variant is overexpressed. Ordinary one-way ANOVAs with multiple comparisons were performed on the data. ∗*p* < 0.05, ∗∗*p* < 0.01; ∗∗∗*p* < 0.001, ∗∗∗∗*p* < 0.0001. Size bar is 500 μM.

To assess how FZD4 regulates retinal vascular development, we used lentiviral transfection to (i) knock down the expression of *FZD4* using shRNA and (ii) express a known disease-causing dominant-negative allele of *FZD4* (*FZD4* p.Met493_Trp494del) (2, 4, 39) in primary human retinal microvascular endothelial cells (hRMECs). To determine the ability of hRMECs to tubularize, a tubulogenesis assay, a mimic of vessel formation and integrity, was performed. The results from this assay showed a large and obvious decrease when FZD4 expression was knocked down by shRNA (Fig. 2*A*). Computational analysis determined an 80% to 90% decrease in the number of tubule junctions, branching intervals, branch length, and mesh number, size, and index as well as a 50 to 75% decrease in total tubule length and total number of branches (Fig. 2*B*, Table S1, Fig. S1). Expression of the FEVR causing *FZD4* p.Met493_Trp494del dominant negative allele in hRMECs also caused a large decrease in retinal endothelial cell tubularization with all parameters decreasing by 80 to 90% (Fig. 2, *C* and *D*). Interestingly, knockdown of FZD4 activity by either *FZD4* shRNA, or expression of the *FZD4* p.Met493_Trp494del dominant negative allele, did not alter growth of hRMECs (Fig. S2). Overall, defective tubularization was observed using two different methods, knockdown of *FZD4*, and expression of a known disease-causing dominant-negative allele of *FZD4*, which strongly supports the idea that decreased FZD4 activity causes defective tubularization in hRMECs.

### The S1PR inhibitor JTE-013 ameliorates the FZD4-mediated primary human retinal microvascular endothelial cell tubularization defects

**Figure 3:**
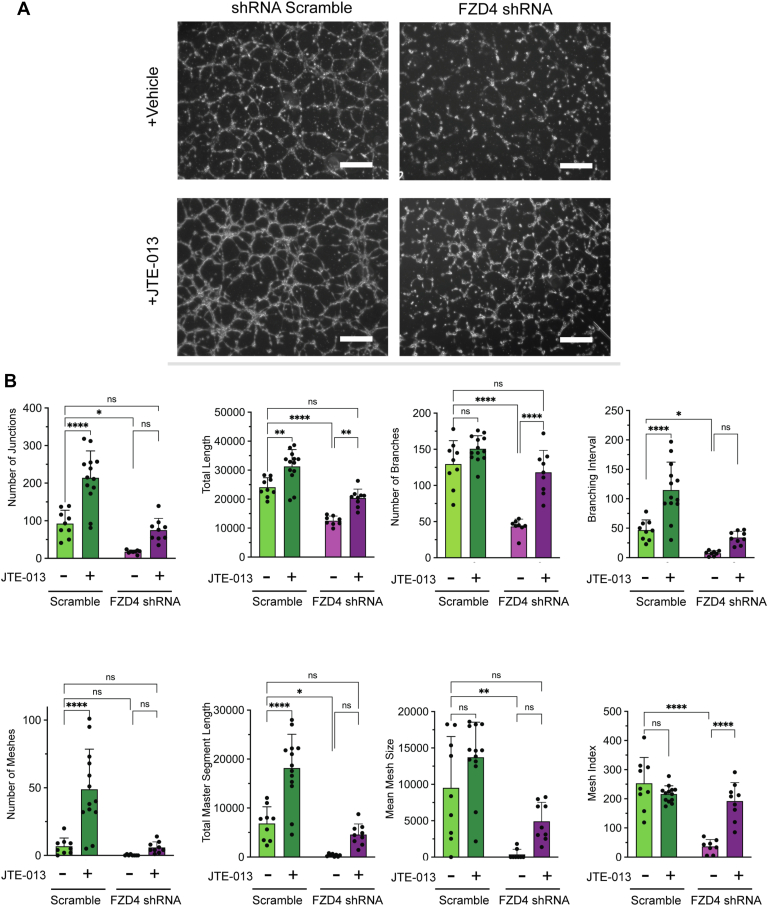
**The S1PR inhibitor JTE-013 rescues *FZD4* mediated primary human retinal microvascular endothelial cell (hRMEC) tubularization defects.***A*, microscopy of vascular formation in the primary hRMECs treated with *FZD4* shRNA and the control (shRNA scramble) with and without JTE-013. Cells were seeded onto a basement membrane and incubated for 6 h at 37 °C prior to imaging. JTE-013 was added at the time of seeding. *B*, parameters measured by the angiogenesis plugin on FIJI and their statistical analysis. Ordinary one-way ANOVAs with multiple comparisons were performed on the data. ∗*p* < 0.05, ∗∗*p* < 0.01; ∗∗∗*p* < 0.001, ∗∗∗∗*p* < 0.0001. Size bar is 500 μM.

S1P signaling is known to affect vascular endothelial cell growth and vessel formation (27, 28, 29, 31, 33, 34, 36, 40). We sought to determine if inhibition of S1PR2 signaling could restore the tubularization defect observed upon FZD4 shRNA expression knockdown. To do so, tubulogenesis assays were performed with hRMECs treated with JTE-013, a well characterized high specificity and high affinity S1PR2 inhibitor (37, 38, 41). JTE-013 treatment of *FZD4* shRNA hRMECs increased every parameter of normal tubularization including junction number, total length, branch number, branching intervals, total segment length, number of meshes, and mesh size and index compared to scramble RNA treated (Fig. 3, *A* and *B*). Indeed, all parameters were increased to the same level as that of hRMECs that were scramble shRNA-treated only, suggesting full tubularization restoration. Interestingly, JTE-013 treatment also increased these same parameters in hRMECs, where FZD4 level was not knocked down by shRNA treatment.

**Figure 4:**
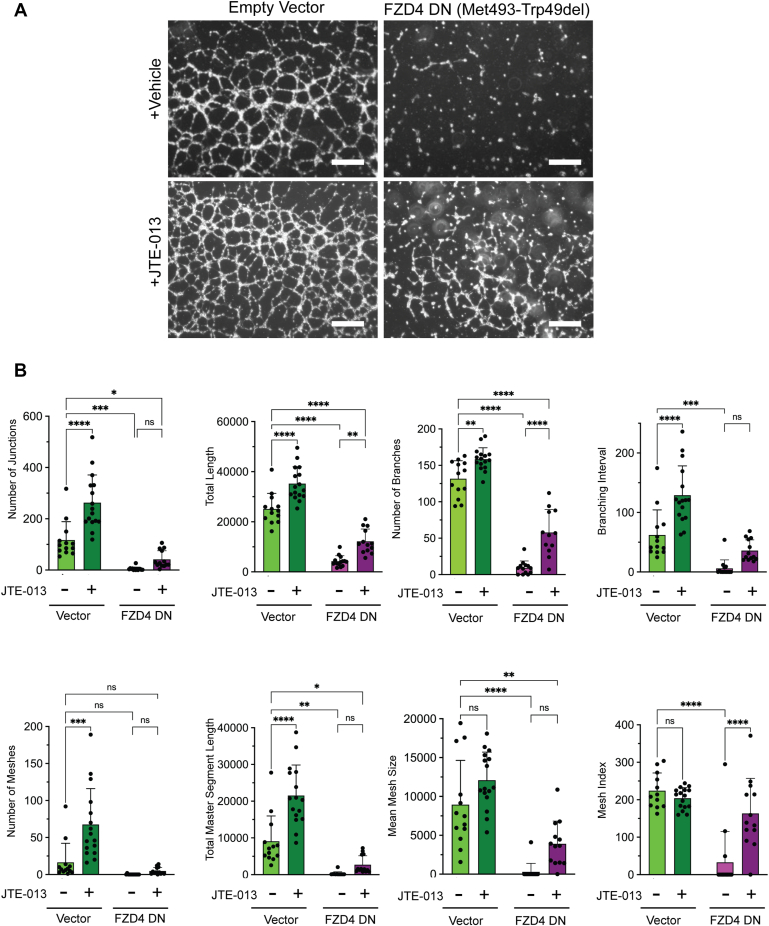
**The S1PR inhibitor JTE-013 rescues primary human retinal microvascular endothelial cell (hRMEC) tubularization defects due to a patient-derived *FZD* dominant negative allele.***A*, microscopy of vascular formation due to expression of the patient-derived dominant negative allele FZD4 p.Met493-Trp49del. (FZD4 DN) in hRMECs and control (empty vector) with and without JTE-013. Cells were seeded onto a basement membrane and incubated for 6 h at 37 °C prior to imaging. JTE-013 was added at the time of seeding. *B*, parameters measured by the angiogenesis plugin on FIJI and their statistical analysis. Ordinary one-way ANOVAs with multiple comparisons were performed on the data. ∗*p* < 0.05, ∗∗*p* < 0.01; ∗∗∗*p* < 0.001, ∗∗∗∗*p* < 0.0001. Size bar is 500 μM.

JTE-013 treatment of hRMECs transduced with the patient-derived dominant negative *FZD4* (*FZD4* p.Met493_Trp494del) allele also increased every parameter of normal tubularization compared to empty vector-treated cells (Fig. 4, *A* and *B*), with the majority of tubularization parameters restored to a similar level as that of hRMECs treated with empty vector. Similar to the FZD4 shRNA knockdown experiments, JTE-013 treatment also increased these tubularization parameters in hRMECs transfected with the empty vector. This chemical genetic effect suggests that S1PR2 inhibition may regulate hRMEC tubularization through a mechanism separate from that of FZD4 signaling.

### Inhibition of S1PR2 signaling ameliorates retinal vascularization in a mouse model of FEVR

**Figure 5:**
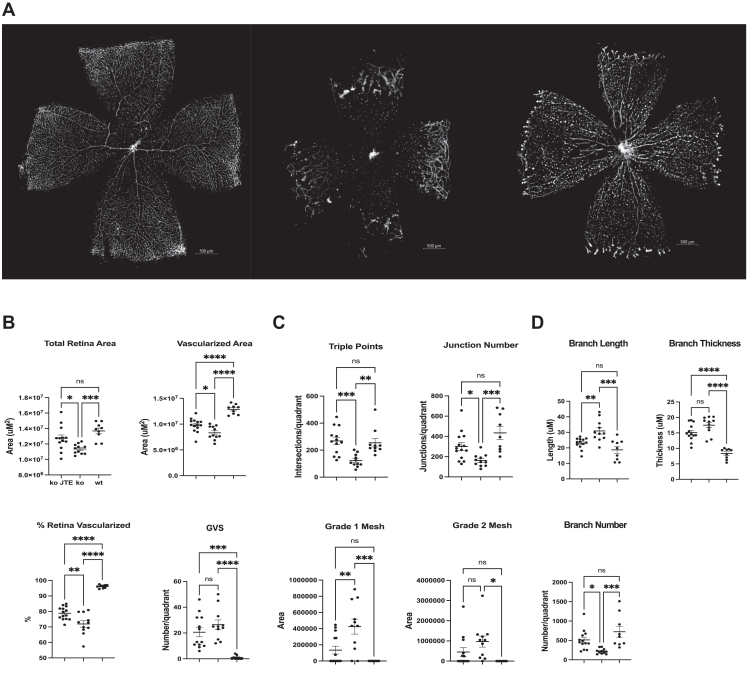
**Amelioration of a mouse FEVR model by S1PR2 inhibition.***A*, Rare variants in the *FZD4* gene cause FEVR in patients. The well characterized mouse model of FEVR (*Fzd4*^*−/−*^ mice) were injected intraperitoneally with the S1PR2 inhibitor JTE-013 at a dose of 1.6 mg/kg once a day from P3 to P14. At P14 retinas were removed, flat mounted, and stained with Alexa Fluor 594 conjugated GS-IB4 lectin to visualize retinal endothelial cells. Retinas were visualized using a Zeiss Axio Imager Z2 microscope containing a digital stage and the MosaiX software program in Axiovision 4.8. All analyses were performed on images captured at 10x magnification with the same image acquisition settings. Representative images are shown. *B–D*, a standardized computational approach that captures and measures structural features in mouse retina flat mounts (Table S2) was used to determine the aspects of retinal vasculature that were ameliorated by treatment with the S1PR2 inhibitor JTE-013. *Fzd4*^*−/−*^ JTE-013 treated (n = 13); *Fzd4*^*−/−*^ vehicle (n = 11); wild type (wt) (n = 9). Ordinary one-way ANOVAs with multiple comparisons were performed on the data. ∗*p* < 0.05, ∗∗*p* < 0.01; ∗∗∗*p* < 0.001, ∗∗∗∗*p* < 0.0001. Size bar is 500 μM.

We sought to determine whether S1PR2 signaling inhibition through JTE-013 treatment could restore retinal vascularization to the well-characterized *Fzd4*^*−/−*^ mouse model of FEVR. Mice were injected once per day intraperitoneally with JTE-013 at 1.6 mg/kg from P3 to P13. At P14, retinas were flat mounted and retinal vascular endothelial cells were visualized by staining with Alexa Fluor 594 conjugated GS-IB4 lectin and analyzed by confocal microscopy and computational image assessment. Subsequent to treatment of *Fzd4*^*−/−*^ mice with JTE-013, there was a large and dramatic increase in retinal vascularization (Fig. 5*A*). Computational determination of the nature of this vascularization showed improvements in all aspects of normal retinal vascularization with total vascularization area, vascular triple junction points, vascular junction number, Grade 1 and Grade 2 mesh, branch length and branch number restored to levels that were statistically similar to wild-type mice (Fig. 5, *B*–*D*, Table S2). In addition, vascularized area, % retina vascularized, glomeruloid vascular structures (GVS) or abnormal microaneurysm-like structures, and branch thickness was significantly improved by JTE-013 treatment of *Fzd4*^*−/−*^ mice compared to vehicle control.

## Discussion

The major implications of our findings are (i) a major contributor to abnormal retinal angiogenesis due to decreased FZD4 activity likely arises from defective vascular formation/integrity, (ii) treatment with the well-characterized S1PR2 inhibitor JTE-013 dramatically improves retinal vascularization in a well-characterized mouse model of FEVR, and (iii) this improvement by JTE-013 was recapitulated in hRMECs, suggesting this improvement is mainly at the level of endothelial cells.

This is the first report we are aware of that links S1PR signaling to the FEVR phenotype, and more importantly that a small (drug-like) molecule can reverse the FEVR phenotype. The cause of defective vascularization of the retina in FEVR is currently unclear; however, the fact that knockdown of FZD4 activity by either shRNA or expression of the patient-derived dominant negative allele in hRMECs causes a dramatic decrease in their capacity to tubularize (but did not alter growth) suggests an inability to vascularize or maintain vascular integrity as a major biological process defective in FEVR.

Our results are consistent with previous work where S1PR signaling has been associated with retinal endothelial cell angiogenesis. S1PR gradients were shown to enable vascular endothelial cell differentiation, enable normal vascular patterning, prevent aberrant neovascularization, and enable normal vascular barrier formation/function (27, 28, 30, 31, 35, 36, 42). Furthermore, in a mouse model of oxygen induced retinopathy, which is meant to mimic retinopathy of prematurity (ROP) in humans, which also results in an inability to vascularize the retinal periphery, activation of S1PR1 signaling or inhibition of S1PR2 signaling using genetic models in mice resulted in more normal retinal vascular patterning (34, 40). Interestingly, rare polymorphisms in FEVR genes, including *FZD4*, predispose infants to more severe ROP (43, 44, 45, 46), implying a degree of overlap in the mechanisms that cause each disease.

Beyond our observation that JTE-013 can restore normal retinal endothelial cell patterning and formation in the absence of FZD4 function, other potential treatments for FEVR have been proposed. Biologics, including a norrin peptide mimetic (47) and a norrin surrogate antibody that increases FZD4-LRP5 interaction (48), have proven effective in the relevant FEVR models. However, these would be limited to specific FEVR genotypes.

Here we show that inhibition of S1PR2 signaling has the potential to treat FEVR. Interestingly, JTE-013 increased tubularization of both control hRMECs and those where FZD4 activity was reduced, implying that an intact FZD4 signaling pathway is not required for JTE-013 to ameliorate FEVR. This type of chemical genetic interaction is generally ascribed to a mechanism by which parallel pathways do not interact biochemically but have the same biological endpoint. The FZD4 pathway is coupled to β-catenin which is thought to then enter the nucleus and drive genes that promote vascularization and angiogenesis through β-catenin interaction with the TCF-LEF transcription factors (3, 5, 13, 14, 15, 17, 18, 20, 23, 24). The genes whose expression is altered by the FZD4 pathway are largely unknown. The S1PR2 pathway is driven primarily *via* G_12/13_ to regulate vascularization and angiogenesis through the Rac and Rho kinase (ROCK) pathways. Both Rac and ROCK regulate the actin network, with Rac thought to signal through p21-activated kinase (PAK) to regulate actin cytoskeleton formation while ROCK increases the phosphorylation of myosin light chain and cofilin which drive the actin filament to stress fiber transition (27, 28, 29, 30, 36, 37). A subject of future work should be defining the precise signaling and biochemical processes by which FZD4 and S1PR2 maintain vascular integrity in the retina to add to the mechanism(s) by which inhibition of S1PR2 inhibition reverses the FEVR disease phenotypes associated with FZD4 pathway inhibition observed here. Future studies could also determine if JTE-013 itself could be moved into the clinic for the treatment of FEVR, and other related conditions including ROP.

## Experimental procedures

### Sex as a biological variable

Our study examined male and female mice with similar findings noted for both sexes.

### Mice

Experiments were carried out using mice bred from *Fzd4*^+/−^ male and female breeders (B6;129-*Fzd4*^*tm1Nat*^/J; The Jackson Laboratory). Mice were injected intraperitoneally with the S1PR2 inhibitor JTE-013 (or excipient control) at a dose of 1.6 mg/kg once a day from P3 to P13. The excipient used for JTE-013 was 85% PBS, 10% KolliPhor EL, 5% DMSO. *Fzd4*^*−/−*^ JTE-013 treated (n = 13); *Fzd4*^*−/−*^ vehicle (n = 11); wild type (wt) (n = 9).

### Mouse genotyping

Mouse genomic DNA was extracted (AccuStart II Mouse Genotyping Kit, Quanta Biosciences) from ear tissue. Mouse *Fzd4* gene primers were obtained from Integrated DNA Technologies, Inc and for the wild type *Fzd4* gene were WT F 5′-TGG AAA GCG TAA TGG TCA AGA TCGG and WT R 5′-AGA ATT CAC CAA TCG GTT AGA ACAC and for the *Fzd4* gene knockout were Mut F 5′-TGT CTG CTA GAT CAG CCT CTG CCG and Mut R 5′-CAT CAA CAT TAA ATG TGA GCG AGT. Mouse genotype was determined by PCR and agar gel analysis.

### Mouse retina isolation and imaging

Mice were euthanized at P14 using a precision vaporizer with induction chamber by administration of 5% isoflurane in oxygen until respiratory arrest occurred (∼1–1.5 min). The chamber was flushed with oxygen and animals were removed and rapid removal of eyes and retinal dissection and flat mounting was performed. The well-established and validated method using Alexa Fluor 594 conjugated GS-IB4 lectin was used to visualize endothelial cells of the vasculature in retina flat mounts as described in previous studies (17, 18, 20, 43, 49). Briefly, retinas were isolated and fixed in Dulbecco's phosphate buffered saline (PBS) containing 0.1 g/l MgCl_2_ and 0.133 g/l CaCl_2_ (Sigma-Aldrich) in 4% paraformaldehyde (Cedarlane) for 1 h at room temperature followed by the addition of PBS containing 0.3% Triton X-100 and 10% goat serum (Sigma-Aldrich) for 1 h at room temperature. To visualize the vascular endothelium, retinas were stained with Alexa Fluor 594-conjugated GS-IB4 lectin (20 μg/ml; Molecular Probes) at 4 °C overnight, washed three times with PBS containing 0.1% Triton X-100 and flat-mounted using Fluoromount-G (Sigma-Aldrich). Retinas were visualized using a Zeiss Axio Imager Z2 microscope containing a digital stage and the MosaiX software program in Axiovision 4.8. All analyses were performed on images captured at 10× magnification with the same image acquisition settings. The parameters analyzed by computational analyses are described in detail in the Table S1. To computationally analyze mouse retina flat mounts retinal images were captured and analyzed using ImageJ software (National Institutes of Health) (50, 51, 52, 53, 54, 55, 56). The scale for each image was calibrated to convert pixel measurements to micrometers by setting the known scale using the Analyze > Set Scale function, after drawing a line over the scale bar with the line tool. Measurements were specified to include area and area fraction, and labels were displayed for each measurement. The total retinal area was demarcated using the polygon selections tool to outline retina outer border, followed by the addition of these selections to the Region of Interest (ROI) manager. Vascularized areas were similarly outlined and measured. Vascular meshwork disorganization within the retinal images was graded based on the extent of vessel fusion and disorder. Grade 1 mesh is characterized by vessel fusion with some ordered structure while Grade 2 mesh area is marked by almost complete disarray of the endothelial cells (49, 50, 51, 52, 53, 55, 56).

Representative regions from the retinal midperiphery were selected, avoiding non-representative areas and those with Grade 1 or 2 vascular disorganization, ROIs were saved and subjected to subsequent image processing which involved conversion to grayscale and contrast enhancement using the CLAHE algorithm. Background noise was reduced using Non-Local Means Denoising and Unsharp Mask filters, followed by Median filtering to remove salt-and-pepper noise. If necessary, the Subtract function was employed for additional noise attenuation. Images were binarized to isolate vascular structures, and particle analysis was conducted to filter out elements smaller than 100 μm^2^, thus refining the representation of the vascular network. Morphological filters were applied to smooth vessel borders. Subsequently, vessel density was quantified based on the binary images. Skeletonization was performed to collapse the vascular network to its centerlines, followed by pruning of branches below a threshold length to remove false branches that were counted by the algorithm due to noise artifacts. The average branch length, branch diameter, and number of junctions and triple points were measured using the Analyze Skeleton function. Glomeruloid vascular structures (GVS) or abnormal microaneurysm-like structures appear as thickened vascular protrusions representing stunted growth of vascular sprouts along the primary mouse retinal vasculature were manually counted within the ROIs. The morphometric analysis was performed using the BoneJ plugin for ImageJ (49, 50, 51, 52, 53, 54, 55, 56). The parameters collected listed Table S2 along with their definitions.

### Cell culture

Human primary retinal microvascular endothelial cells (hRMECs,) were utilized. Cell lines were routinely tested for *mycoplasma* by PCR analysis. Site-directed mutagenesis of pLenti-C-mGFP-P2A-Puro-FZD4 was used to create the known *FZD4* dominant negative disease-causing allele *FZD4* p.Met493-Trp49del (pLenti-C-mGFP-P2A-Puro-FZD4 DN) for subsequent expression in cells. Human shRNA Plasmid Kit (Origene, catalog number TL312883) was used for shRNA knockdown of *FZD4* expression. The shRNAs used were:

FZD4A-tcttggcaactttgcattcacacagatta;taatctgtgtgaatgcaaagttgccaaga;

FZD4B–gtataagccagcatcatagccacacttgag;gctcaagtgtggctatgatgctggcttata;

FZD4C–gcatcttggtcacgttgtagccgaggttctg;agaacctcggctacaacgtgaccaagatg;

FZD4D – gcagaccaaatccacatgcctgaagtgatg; gcagaccaaatccacatgcctgaagtgatg.

The hRMECs were cultured in Lonza Clonetics EGM 2 MV SingleQuots media (catalog number CC-4147) at 37 °C in 5% CO_2_. The cells were not used past passage 14. To transduce the hRMECs for the knockdown of *FZD4* by shRNA, or the expression of FZD4 p.Met493-Trp49del by pLenti-C-mGFP-P2A-Puro-FZD4 DN, the OriGene application guide was followed. Positive transfection was determined *via* the percentage of hRMECs expressing GFP. The hRMEC transfection was routinely over 80% as determined by microscopy for GFP-positive cells. FZD4 knockdown and FZD4 p.Met493-Trp49del expression were confirmed by Western blot. Two controls were used, scrambled shRNA hRMECs as a control for *FZD4* shRNA and an empty vector as a control for pLenti-C-mGFP-P2A-Puro-FZD4 DN.

### Tubulogenesis assay

For the tubulogenesis assay, 6-well plates were coated with 600 μl of a 2:1 ratio of Geltrex and Lonza Clonetics EBM-2 media, catalog numbers A1413302 and C-3156, respectively. This layer mimics the basement membrane matrix and was allowed to solidify for 1 h at 37 °C. The hRMEC cells were seeded in the wells at a density of 200,000 cells per well and the total final volume in each well was 2.4 ml. At the time of seeding, cells were treated with either DMSO as the vehicle control or with 20 μM of JTE-013. The cells were then incubated at 37 °C for 6 h prior to imaging. Images were taken on an Invitrogen EVOS M5000 Imaging System at 4X magnification using brightfield phase contrast. Images were analyzed using the angiogenesis analysis plug within the FIJI (FIJI is just Image J) software. Multiple images for each condition were captured and used for analysis. The tubulogenesis parameters collected from the FIJI angiogenesis plugin are listed in Table S1 along with their definitions, and Fig. S1 illustrates these parameters. Ordinary one-way ANOVAs with multiple comparisons were performed on the data. The experiment was repeated three times.

### Cell migration assay

For the cell migration assay, cells were seeded in the ibidi Culture-Insert 2 Well in a 35 mm μ-Dish (catalog number 81176). The hRMECs were seeded at 125,000 cells/ml. Total volume in each well of the insert was 70 μl. Cells were incubated for 48 h at 37 °C before insert removal. Inserts were removed and the dish was washed three times with 1 ml Lonza Clonetics EGM 2 MV SingleQuots media to remove non-adherent cells. Once washed, fresh 2 ml Lonza Clonetics EGM 2 MV SingleQuots media was added to each dish, with either vehicle control or 2 μM JTE-013 (5 ng of mitomycin C was added to inhibit cell proliferation) and images were taken using a light microscope at 0, 4, and 8 h at 100X magnification. Three images were taken of each dish for analysis. Images were analysed using the wound healing tool on FIJI. The percentage of area covered by cells in the images was measured. Ordinary one-way ANOVAs with multiple comparisons were performed on the data. The experiment was repeated three times.

### Statistical analysis

Data were analyzed using GraphPad Prism software (v.10.4.0). Data are represented as mean ± SD. Ordinary one-way ANOVAs with multiple comparisons were performed on the data. For all analysis *p* < 0.05 was considered significant.

### Study approval

All procedures were performed in accordance with the Guide to Care and Use of Experimental Animals by the Canadian Council on Animal Care and approved by the Research Ethics Board for Laboratory Animal Research at Dalhousie University.

## Data availability

All the data are contained within the manuscript or the supporting information.

## Supporting information

This article contains supporting information.

## Conflict of interest

The authors declare that they have no conflicts of interest with the contents of this article.

## References

[1] Robitaille J.M., Guernsey D.L., Traboulsi E.I., Traboulsi E.I. (2011). Norrie disease and Other Developmental Retinal Vascular Disorders in Genetic Diseases of the Eye.

[2] Robitaille J., MacDonald M.L., Kaykas A., Sheldahl L.C., Zeisler J., Dube M.P. (2002). Mutant frizzled-4 disrupts retinal angiogenesis in familial exudative vitreoretinopathy. Nat. Genet..

[3] Robitaille J.M., G D.L., Traboulsi E.I., Traboulsi E.I. (2011). Norrie disease and Other Developmental Retinal Vascular Disorders in Genetic Diseases of the Eye.

[4] Tao T., Xu N., Li J., Li H., Qu J., Yin H. (2021). Ocular features and mutation spectrum of patients with familial exudative Vitreoretinopathy. Invest. Ophthalmol. Vis. Sci..

[5] Selvam S., Kumar T., Fruttiger M. (2018). Retinal vasculature development in health and disease. Prog. Retin. Eye Res..

[6] Chen Z.Y., Battinelli E.M., Fielder A., Bundey S., Sims K., Breakefield X.O. (1993). A mutation in the Norrie disease gene (NDP) associated with X-linked familial exudative vitreoretinopathy. Nat. Genet..

[7] Poulter J.A., Ali M., Gilmour D.F., Rice A., Kondo H., Hayashi K. (2016). Mutations in TSPAN12 cause autosomal-dominant familial exudative vitreoretinopathy. Am. J. Hum. Genet..

[8] van der Ende S.R., Meyers B.S., Capasso J.E., Sasongko M., Yonekawa Y., Pihlblad M. (2022). Severe familial exudative vitreoretinopathy, congenital hearing loss, and developmental delay in a child with biallelic variants in FZD4. JAMA Ophthalmol..

[9] Jiao X., Ventruto V., Trese M.T., Shastry B.S., Hejtmancik J.F. (2004). Autosomal recessive familial exudative vitreoretinopathy is associated with mutations in LRP5. Am. J. Hum. Genet..

[10] Toomes C., Bottomley H.M., Jackson R.M., Towns K.V., Scott S., Mackey D.A. (2004). Mutations in LRP5 or FZD4 underlie the common familial exudative vitreoretinopathy locus on chromosome 11q. Am. J. Hum. Genet..

[11] Hoem G., Pastore A., Bratland E., Christoffersen T., Stornaiuolo M., Douzgou S. (2024). Severe isolated exudative vitreoretinopathy caused by biallelic FZD4 variants. Clin. Genet..

[12] van der Ende S., Bedard K., Wallace K., Mackley M.P., Nightingale M., Gaston D. (2025). Gene variant spectrum in probands with familial exudative vitreoretinopathy using an expanded panel. Invest. Ophthalmol. Vis. Sci..

[13] Junge H.J., Yang S., Burton J.B., Paes K., Shu X., French D.M. (2009). TSPAN12 regulates retinal vascular development by promoting Norrin- but not Wnt-induced FZD4/beta-catenin signaling. Cell.

[14] Ke J., Harikumar K.G., Erice C., Chen C., Gu X., Wang L. (2013). Structure and function of Norrin in assembly and activation of a frizzled 4-Lrp5/6 complex. Genes Dev..

[15] Lai M.B., Zhang C., Shi J., Johnson V., Khandan L., McVey J. (2017). TSPAN12 is a norrin Co-receptor that amplifies Frizzled4 ligand selectivity and signaling. Cell Rep..

[16] Qian Y., Ma Z., Xu Z., Duan Y., Xiong Y., Xia R. (2024). Structural basis of Frizzled 4 in recognition of dishevelled 2 unveils mechanism of WNT signaling activation. Nat. Commun..

[17] Wang Y., Rattner A., Zhou Y., Williams J., Smallwood P.M., Nathans J. (2012). Norrin/Frizzled4 signaling in retinal vascular development and blood brain barrier plasticity. Cell.

[18] Xu Q., Wang Y., Dabdoub A., Smallwood P.M., Williams J., Woods C. (2004). Vascular development in the retina and inner ear: control by Norrin and Frizzled-4, a high-affinity ligand-receptor pair. Cell.

[19] Yang M., Li S., Huang L., Zhao R., Dai E., Jiang X. (2022). CTNND1 variants cause familial exudative vitreoretinopathy through the Wnt/cadherin axis. JCI Insight.

[20] Ye X., Wang Y., Cahill H., Yu M., Badea T.C., Smallwood P.M. (2009). Norrin, frizzled-4, and Lrp5 signaling in endothelial cells controls a genetic program for retinal vascularization. Cell.

[21] Zhang C., Lai M.B., Pedler M.G., Johnson V., Adams R.H., Petrash J.M. (2018). Endothelial cell-specific inactivation of TSPAN12 (Tetraspanin 12) reveals pathological consequences of barrier defects in an otherwise intact vasculature. Arterioscler. Thromb. Vasc. Biol..

[22] Paes K.T., Wang E., Henze K., Vogel P., Read R., Suwanichkul A. (2011). Frizzled 4 is required for retinal angiogenesis and maintenance of the blood-retina barrier. Invest. Ophthalmol. Vis. Sci..

[23] Yang S., Wu Y., Xu T.H., de Waal P.W., He Y., Pu M. (2018). Crystal structure of the Frizzled 4 receptor in a ligand-free state. Nature.

[24] Ye X., Wang Y., Nathans J. (2010). The Norrin/Frizzled4 signaling pathway in retinal vascular development and disease. Trends Mol. Med..

[25] Fei P., Liu Z., He L., Li N., Xu L., Zhang M. (2021). Early detection of ocular abnormalities in a Chinese multicentre neonatal eye screening programme-1-year result. Acta. Ophthalmol..

[26] Alshaikh R.A., Ryan K.B., Waeber C. (2022). Sphingosine 1-phosphate, a potential target in neovascular retinal disease. Br. J. Ophthalmol..

[27] Cartier A., Hla T. (2019). Sphingosine 1-phosphate: lipid signaling in pathology and therapy. Science.

[28] Gaengel K., Niaudet C., Hagikura K., Lavina B., Muhl L., Hofmann J.J. (2012). The sphingosine-1-phosphate receptor S1PR1 restricts sprouting angiogenesis by regulating the interplay between VE-cadherin and VEGFR2. Dev. Cell.

[29] Hannun Y.A., Obeid L.M. (2018). Sphingolipids and their metabolism in physiology and disease. Nat. Rev. Mol. Cell Biol..

[30] Jung B., Obinata H., Galvani S., Mendelson K., Ding B.S., Skoura A. (2012). Flow-regulated endothelial S1P receptor-1 signaling sustains vascular development. Dev. Cell.

[31] Lee M.J., Thangada S., Claffey K.P., Ancellin N., Liu C.H., Kluk M. (1999). Vascular endothelial cell adherens junction assembly and morphogenesis induced by sphingosine-1-phosphate. Cell.

[32] Porter H., Qi H., Prabhu N., Grambergs R., McRae J., Hopiavuori B. (2018). Characterizing sphingosine kinases and sphingosine 1-Phosphate receptors in the Mammalian eye and Retina. Int. J. Mol. Sci..

[33] Shiwani H.A., Elfaki M.Y., Memon D., Ali S., Aziz A., Egom E.E. (2021). Updates on sphingolipids: spotlight on retinopathy. Biomed. Pharmacother..

[34] Skoura A., Sanchez T., Claffey K., Mandala S.M., Proia R.L., Hla T. (2007). Essential role of sphingosine 1-phosphate receptor 2 in pathological angiogenesis of the mouse retina. J. Clin. Invest..

[35] Weigel C., Bellaci J., Spiegel S. (2023). Sphingosine-1-phosphate and its receptors in vascular endothelial and lymphatic barrier function. J. Biol. Chem..

[36] Yanagida K., Engelbrecht E., Niaudet C., Jung B., Gaengel K., Holton K. (2020). Sphingosine 1-Phosphate receptor signaling establishes AP-1 gradients to allow for retinal endothelial cell specialization. Dev. Cell.

[37] Zhang G., Yang L., Kim G.S., Ryan K., Lu S., O'Donnell R.K. (2013). Critical role of sphingosine-1-phosphate receptor 2 (S1PR2) in acute vascular inflammation. Blood.

[38] Kim G.S., Yang L., Zhang G., Zhao H., Selim M., McCullough L.D. (2015). Critical role of sphingosine-1-phosphate receptor-2 in the disruption of cerebrovascular integrity in experimental stroke. Nat. Commun..

[39] van der Ende S., Gaston D., Nightingale M., Wallace K., Bedard K., Leblanc M. (2025). Gene variant spectrum in probands with familial exudative vitreoretinopathy. IOVS.

[40] Niaudet C., Jung B., Kuo A., Swendeman S., Bull E., Seno T. (2023). Therapeutic activation of endothelial sphingosine-1-phosphate receptor 1 by chaperone-bound S1P suppresses proliferative retinal neovascularization. EMBO. Mol. Med..

[41] Xu Q., Chen J., Zhu Y., Xia W., Liu Y., Xu J. (2021). JTE-013 alleviates inflammatory injury and endothelial dysfunction induced by sepsis in vivo and in vitro. J. Surg. Res..

[42] McGuire P.G., Rangasamy S., Maestas J., Das A. (2011). Pericyte-derived sphingosine 1-phosphate induces the expression of adhesion proteins and modulates the retinal endothelial cell barrier. Arterioscler. Thromb. Vasc. Biol..

[43] Ngo M.H., Borowska-Fielding J., Heathcote G., Nejat S., Kelly M.E., McMaster C.R. (2016). Fzd4 haploinsufficiency delays retinal revascularization in the mouse model of oxygen induced retinopathy. PLoS One.

[44] Ells A., Guernsey D.L., Wallace K., Zheng B., Vincer M., Allen A. (2010). Severe retinopathy of prematurity associated with FZD4 mutations. Ophthalmic Genet..

[45] Kondo H., Kusaka S., Yoshinaga A., Uchio E., Tawara A., Tahira T. (2013). Genetic variants of FZD4 and LRP5 genes in patients with advanced retinopathy of prematurity. Mol. Vis..

[46] Dailey W.A., Gryc W., Garg P.G., Drenser K.A. (2015). Frizzled-4 variations associated with retinopathy and intrauterine growth retardation: a potential marker for prematurity and retinopathy. Ophthalmology.

[47] Chidiac R., Abedin M., Macleod G., Yang A., Thibeault P.E., Blazer L.L. (2021). A Norrin/Wnt surrogate antibody stimulates endothelial cell barrier function and rescues retinopathy EMBO. Mol. Med..

[48] Ding J., Lee S.J., Vlahos L., Yuki K., Rada C.C., van Unen V. (2023). Therapeutic blood-brain barrier modulation and stroke treatment by a bioengineered FZD(4)-selective WNT surrogate in mice. Nat. Commun..

[49] Adini I., Ghosh K. (2015). Mouse retinal whole mounts and quantification of vasculature protocol. Bio. Protoc..

[50] Karperien A.L., Jelinek H.F. (2024). ImageJ in computational fractal-based neuroscience: pattern extraction and translational Research. Adv. Neurobiol..

[51] Schroeder A.B., Dobson E.T.A., Rueden C.T., Tomancak P., Jug F., Eliceiri K.W. (2021). The ImageJ ecosystem: Open-source software for image visualization, processing, and analysis. Protein Sci..

[52] Corliss B.A., Doty R.W., Mathews C., Yates P.A., Zhang T., Peirce S.M. (2020). REAVER: a program for improved analysis of high-resolution vascular network images. Microcirculation.

[53] Corliss B.A., Mathews C., Doty R., Rohde G., Peirce S.M. (2019). Methods to label, image, and analyze the complex structural architectures of microvascular networks. Microcirculation.

[54] Domander R., Felder A.A., Doube M. (2021). BoneJ2 - refactoring established research software. Wellcome Open Res..

[55] Milde F., Lauw S., Koumoutsakos P., Iruela-Arispe M.L. (2013). The mouse retina in 3D: quantification of vascular growth and remodeling. Integr. Biol. (Camb).

[56] Zudaire E., Gambardella L., Kurcz C., Vermeren S. (2011). A computational tool for quantitative analysis of vascular networks. PLoS One.

