# Supplementary material for: Sphingosine-1-phosphate receptor 2 inhibition ameliorates familial exudative vitreoretinopathy models
Source: J Biol Chem. 2025 Dec 23;302(2):111107. doi: 10.1016/j.jbc.2025.111107 (PMC12830201; doi:10.1016/j.jbc.2025.111107)
Supplement: Supporting Figures and Tables [file mmc1.pdf]

Supplemental Data

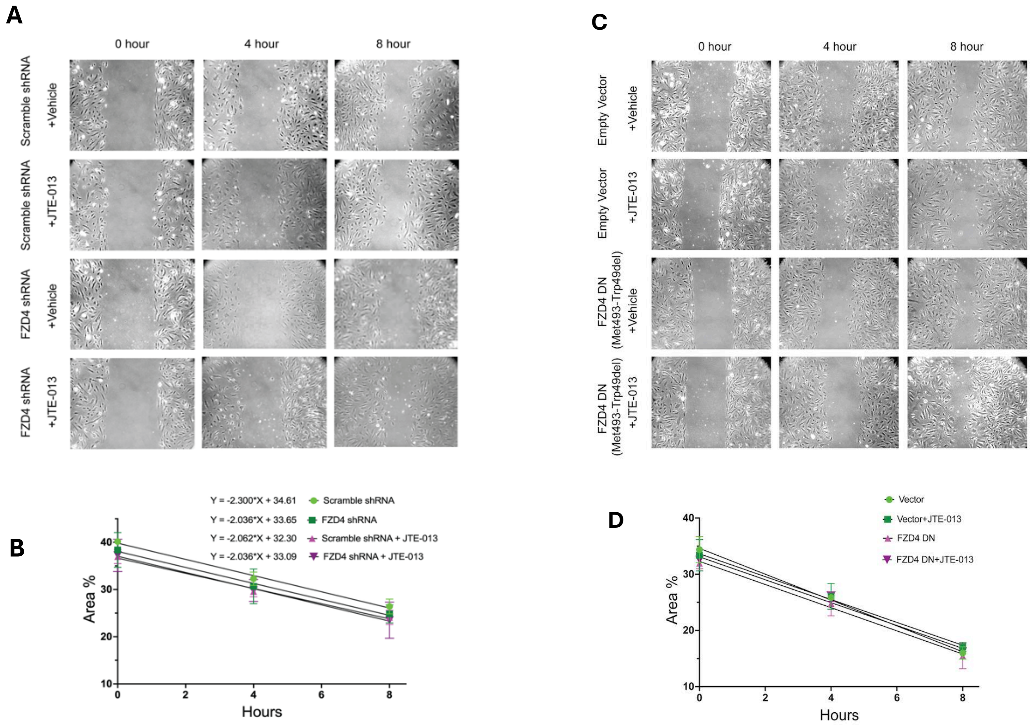

Figure S1. (**A,C)** Microscopy of cell migration in hRMECs treated with *FZD4* shRNA or expression of the patient-derived dominant negative allele *FZD4* p.Met493-Trp49del. (FZD4 DN) in the presence and absence of JTE-013. Cells were seeded in a two well insert with a divider. The divider was removed, and images were taken at three time points; 0 hr, 4 hr, and 8 hr. JTE-013 was added at the time of insert removal. (**B**,**D**) The gap between the two areas of cells was measured at each time point using the wound healing plugin on FIJI. Ordinary one-way ANOVAs with multiple comparisons were performed on the data. There was no significant difference between any treatment.

Table S1. Definitions of the computational tubulogenesis parameters examined in human primary microvascular endothelial cells

Feature	Definition
Junction	Node connecting three or more segments and branches
Master Junction	Node connecting three or more segments only
Branch	Vessel connecting one junction and an extremity
Segment	Vessel connecting two junctions
Master segment	Vessel connecting two master junctions
Total Master Segment Length	Sum of the length of the master segments
Branching Interval	Mean distance between branches in the network.
Mesh	The area enclosed by master segments and master junctions.
Mesh Size	Mean size of the individual meshes
Mesh Index	Mean distance between master junctions

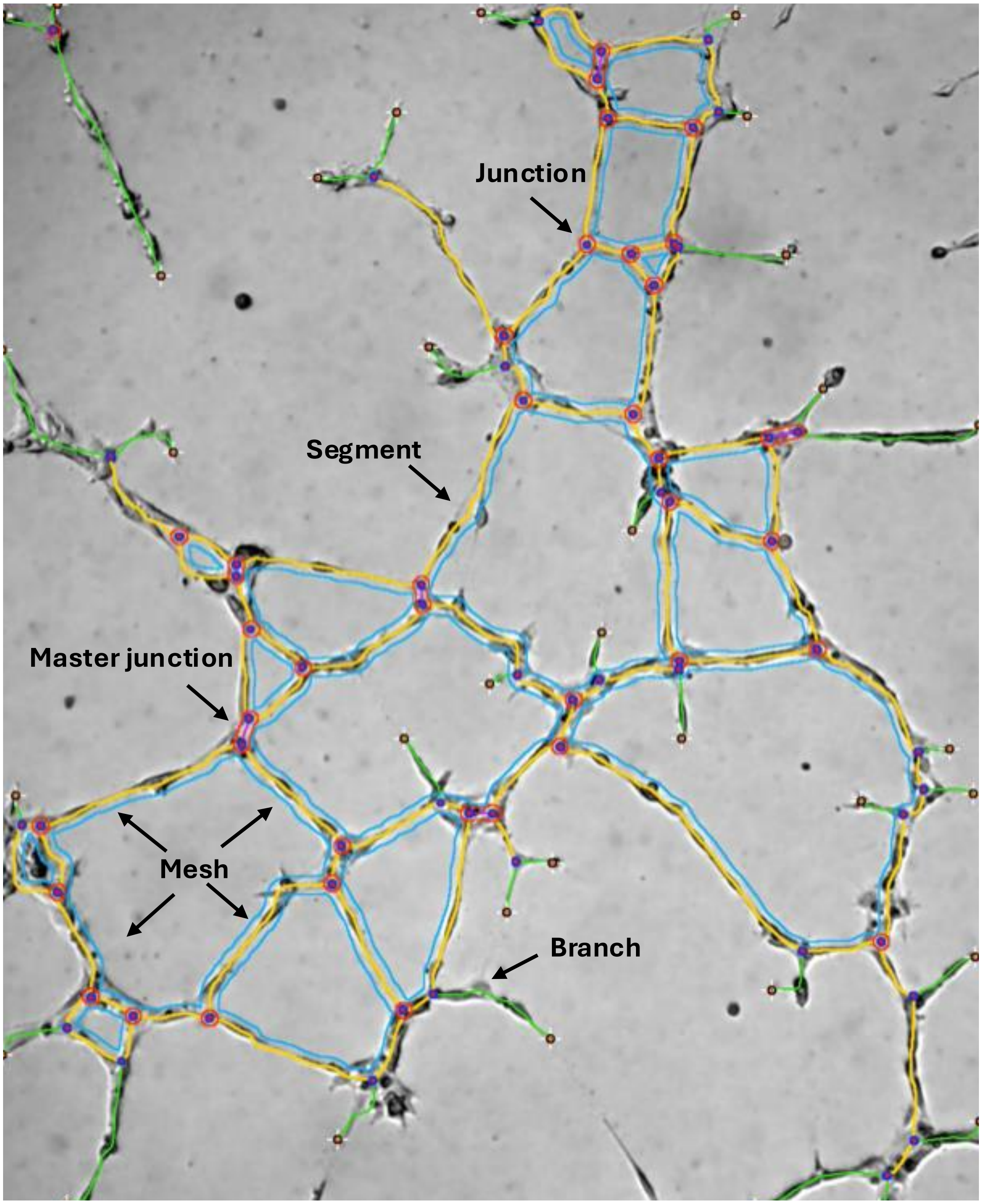

Figure S2. Illustration of the parameters described in Table S1. Red/blue dots are junctions, red/blue dots with an extra red ring are master junctions, green is branches, yellow is segments, and blue outline is a mesh.

Table S2. Definitions of retina flat mount computational parameters examined in mice

Parameter	Definition
Total Retina Area	Total area of the whole retina
Vascularized Area	Total area of the retina that is vascularized
% Vascularized Area	Proportion of total retinal area with vessels
Glomeruloid Vascular Structures	Glomeruloid vascular structures (GVS) or abnormal microaneurysm-like structures
Junction Number	Number of branching points in skeletonized network
Triple Points	Number of junctions between vessel branches where there are three vessels
Grade 1 Mesh	Vessels appear to be fused together but there is still some ordered structure
Grade 2 Mesh	Almost completely disordered mass of endothelial cells
Branch Length	Average branch length
Branch Thickness	Average vessel diameter
Branch Number	Total number of branches
